# Transarterial Embolization for Hepatocellular Carcinoma: A Comparison between Nonspherical PVA and Microspheres

**DOI:** 10.1155/2015/435120

**Published:** 2015-08-27

**Authors:** Leandro Armani Scaffaro, Cleber Dario Pinto Kruel, Steffan Frosi Stella, Gabriela Leal Gravina, Geraldo Machado Filho, Carlos Podalirio Borges de Almeida, Luiz Cezar Pontes Fonseca Pinto, Mario Reis Alvares-da-Silva, Cleber Rosito Pinto Kruel

**Affiliations:** ^1^Radiology Division, Hospital de Clínicas de Porto Alegre (HCPA), Porto Alegre, RS, Brazil; ^2^Surgery Division, Hospital de Clínicas de Porto Alegre (HCPA), Porto Alegre, RS, Brazil; ^3^Institute for Health Technology Assessment, Universidade Federal do Rio Grande do Sul (UFRGS), Porto Alegre, RS, Brazil; ^4^School of Medicine, Universidade Federal do Rio Grande do Sul (UFRGS), Porto Alegre, RS, Brazil; ^5^Gastroenterology Division, Hospital de Clínicas de Porto Alegre (HCPA), Porto Alegre, RS, Brazil

## Abstract

Transarterial chemoembolization (TACE) and transarterial embolization (TAE) have improved the survival rates of patients with unresectable hepatocellular carcinoma (HCC); however, the optimal TACE/TAE embolic agent has not yet been identified. The aim of this study was to compare the effect of two different embolic agents such as microspheres (ME) and polyvinyl alcohol (PVA) on survival, tumor response, and complications in patients with HCC submitted to transarterial embolization (TAE). Eighty HCC patients who underwent TAE between June 2008 and December 2012 at a single center were retrospectively studied. A total of 48 and 32 patients were treated with PVA and ME, respectively. There were no significant differences in survival (*P* = 0.679) or tumoral response (*P* = 0.369) between groups (PVA or ME). Overall survival rates at 12, 18, 24, 36, and 48 months were 97.9, 88.8, 78.9, 53.4, and 21.4% in the PVA-TAE group and 100, 92.9, 76.6, 58.8, and 58% in the ME-TAE group (*P* = 0.734). Patients submitted to TAE with ME presented postembolization syndrome more frequently when compared with the PVA group (*P* = 0.02). According to our cohort, the choice of ME or PVA as embolizing agent had no significant impact on overall survival.

## 1. Introduction

Hepatocellular carcinoma (HCC) is the sixth most common cancer worldwide and the third leading cause of cancer-related death [1]. Unfortunately, potentially curative options are applicable in a minority of HCC patients [2]. Locoregional treatments, such as transarterial chemoembolization (TACE) and transarterial embolization (TAE), have been used as the first-line treatment for intermediate stage HCC patients with proven survival benefits [3–6].

The most widely employed embolizing agents are nonspherical polyvinyl alcohol (PVA) and, more recently, calibrated hydrophilic microspheres (ME) [7, 8]. Despite the worldwide acceptance of TACE and TAE as important tools in the treatment of unresectable HCC, there is considerable heterogeneity in relation to the type of particle, the chemotherapy drugs used, and the schedule of administration. Embolizing particles have been used according to the preference of each physician, and the choice for a particular embolizing agent has not been demonstrated to have a significant impact in overall survival [9]. As a matter of fact, there is no consensus regarding the optimal guidelines for TACE or TAE, especially with respect to which embolizing agent is ideal in a given situation [10].

To provide further insight, we performed a retrospective analysis of patients undergoing TAE with two different embolizing agents (PVA and ME). Equivalency between treatments was evaluated based on overall survival, local tumor response, and incidence of postembolization syndrome (PES).

## 2. Material and Methods

We retrospectively analyzed a historical cohort of patients older than 18 years with the American Association for the Study of Liver Diseases (AASLD) diagnosed HCC who were treated from June 2008 to December 2012 at the Gastroenterology Division, Hepatology Unit of the institution. The study was approved by the Local Ethics Committee (Institutional Review Board-equivalent), and the requirement to obtain informed consent was waived for the retrospective analysis of existing data.

Transarterial embolization was indicated in patients with BCLC A having nodules greater than 3 cm or nodules that cannot be safely accessed percutaneously by ethanol or radiofrequency ablation and in patients with BCLC B or BCLC C with no signs of extrahepatic disease [1].

Patients with BCLC D or BCLC C with evidence of extrahepatic disease, portal vein thrombosis (or thrombosis of one of its branches), or hepatofugal portal flow were excluded. Patients with a definitive diagnosis of extrahepatic metastasis, difficult-to-control ascites, other active malignant diseases, or the following laboratorial anomalies were also excluded: serum creatinine above 1.5 mg/dL, total bilirubin above 4.0 mg/dL, platelet count lower than 50,000/mm^3^, and a prothrombin activity less than 50%.

During this period, 80 patients were treated with PVA or ME-TAE. The choice of the embolizing agent was made according to the agent's availability at the time of the procedure. Once the data were obtained, the patients were divided into two groups: the PVA group (TAE with PVA) and the ME group (TAE with ME).

### 2.1. Procedure

TAE was performed under sedation by the same interventional radiologist through a common femoral access point. Selective catheterization and arteriogram of the celiac trunk and of the superior mesenteric artery were performed with a Cobra or Mikaelson 5 F catheter. The hepatic artery was selectively catheterized, followed by a superselective feeding branch tumor catheterization with a 2.8 F microcatheter (Progreat, Terumo). In the PVA group, PVA (Cook, Bloomington, USA) was selectively injected into the feeding artery at the most distal location possible. In the ME group, this selective injection was performed with ME-embospheres (Biosphere Medical, Rockland, USA). In both groups, the particle sizes were 100–300 *μ*m for tumors up to 5 cm and 300–500 *μ*m for tumors equal to or larger than 5 cm.

Patients with a previous history of biliary manipulation received piperacillin/tazobactam to minimize the risk of infection. Antiemetics or analgesics were not given preemptively.

We used the modified Response Evaluation Criteria in Solid Tumors (mRECIST) to assess the tumoral response to TAE, which determines the response based on the enhancement pattern during the arterial phase of the dynamic study [11]. The tumor response was defined as follows: (a) complete response: complete disappearance of the enhancement of the target lesion(s) during the arterial phase; (b) partial response: a reduction of at least 30% in the sum of the maximum diameters of the viable lesion(s); (c) progressive disease: an increase of at least 20% in the sum of the maximum diameters of the viable lesion(s); and (d) stable disease: when the criteria of the two classifications above are not fulfilled [11].

The effects related to PES were analyzed according to the Southwest Oncology Group Criteria (SOGC) [5], which define PES when there are at least two points (Table 1). PES symptoms were analyzed based on the first TAE in each patient. Possible complications related to TAE are hematoma, liver failure, acute cholecystitis, liver abscess, or death within 30 days of the procedure.

### 2.2. Statistical Analysis

Categorical variables were described using frequencies and percentages. Quantitative variables with symmetric distributions were expressed by their mean values and standard deviations; those with asymmetric distributions were described by the median and interquartile interval. The chi-squared test or Fisher's exact test was used to compare the categorical variables. Quantitative variables with symmetric distributions between groups were compared using Student's *t*-test for independent samples. Variables with asymmetric distributions were compared between the groups using the Mann-Whitney *U* test.

The period between the date of the first embolization and death or last follow-up was used for the survival assessment. The survival rates were calculated using the Kaplan-Meier method, and the groups were compared using the log-rank test. Cox regression was used in order to control for the variables gender and age, which are frequently related to survival.

Calculations were performed using SPSS software (version 19.0). The statistical significance was set at 0.05.

## 3. Results

A total of 80 patients were enrolled in the analysis. Forty-eight patients were treated with PVA, and ME was used in 32 patients. There were 8 liver transplants and 1 lost during the follow-up period in the PVA group, while there were 5 liver transplants and 1 lost in the ME group. The mean numbers of treatment sessions were 2.3 for the group embolized with ME and 2.1 for the group in which PVA was used (*P* = 0.19). A summary of baseline characteristics of the two patient groups is reported in Table 2. There were no significant differences at baseline with respect to demographic data, staging, laboratory characteristics, and HCC characteristics.

Regarding complications, hematoma at the puncture site was detected in 3 patients (6.5%) in the PVA group and 1 patient (3.2%) in the ME group. Decompensated ascites after TAE was noted in 5 patients (11.1%) in the PVA group and in 1 patient (3.2%) in the ME group. Only one patient, from the ME group, developed a liver abscess. No acute cholecystitis or death within 30 days after the procedure was observed in either group.

**Figure 1:**
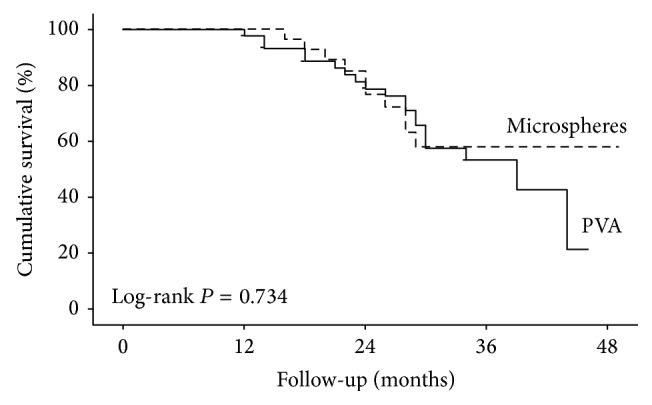
Kaplan-Meier survival curves. PVA: polyvinyl alcohol.

The survival means and medians were 34.3 and 39 months in the PVA group (minimum 25.1 months; maximum 52 months) and 38.1 and 39 months in the ME group (minimum 27.6 months; maximum 50.4 months), and no significant difference was demonstrated between the groups (*P* = 0.679).  Figure 1 presents the Kaplan-Meier curves of the two groups.

The survival rates at 12, 18, 24, 36, and 48 months were 97.9%, 88.8%, 78.9%, 53.4%, and 21.4%, respectively, for the PVA group and 100%, 92.9%, 76.6%, 58.8%, and 58%, respectively, for the ME group, with no differences between the two groups (*P* = 0.734). Even after controlling for gender and age, no significant difference was observed between the groups regarding patient survival (Hazard ratio: 0.847; confidence interval 95%: 0.383–1.877) (*P* = 0.683).

There were no significant differences between the groups with respect to tumoral response according to the mRECIST, as shown in Table 3. Most cases had a partial response after TAE (23 patients (47.9%) in the PVA group and 18 patients (56.3%) in the ME group).

The incidence of PES, defined by the presence of 2 or more points according to the SOGC, was significantly higher for the ME group (56.2%) compared with PVA group (29.1%) (*P* = 0.02).  Table 4 depicts the distribution of PES in the groups.

## 4. Discussion

Due to the unique arterial supply, HCC is susceptible to transcatheter intra-arterial therapies such as TACE and TAE. However, despite its widespread use, TACE and TAE remain as unstandardized procedures, with variation in type and size of embolizing particles, type and dose of chemotherapy, and interval between therapies. Studies comparing TAE and TACE have failed to demonstrate the superiority of one specific strategy over the other [11–13]. In spite of that, the European Association for the Study of Liver Diseases and also the BCLC algorithm have discouraged the use of TAE [14]. In contrast with the European policy, and according to the American Association for the Study of Liver Diseases guidelines [15], TAE has been used in our service as an economic alternative for local control of HCC.

However, the debate about the most appropriate protocol for treating HCC with transcatheter intra-arterial therapy is not restricted to the addition of the chemotherapeutic agent. The selection for the most appropriate embolic particle is still a matter of debate. Permanent or semipermanent arterial occlusion can be achieved with different agents such as Gelfoam, PVA, ME, and more recently drug-eluting beads (DEB-TACE). Nevertheless, there is little evidence in the literature comparing the efficiency between different embolizing agents especially for TAE [16]. Although Gelfoam has been considered as a suboptimal agent, due to the large size of the particles (1 mm) and temporary occlusion of the feeding arteries that only lasts for 2 weeks, similar survival rates have been demonstrated when Gelfoam was compared with PVA in HCC patients treated with TACE [16, 17]. Recently, a randomized controlled study showed no significant difference in 1-year survival when DEB-TACE was compared with bland embolization with ME [12]. Thus, it seems that most of the antitumoral effect is caused by local ischemia, which can be achieved with different embolizing particles.

Our results showed no significant difference in survival for the groups treated with TAE using PVA or ME. The survival rates at 12, 24, and 36 months were 97%, 88%, and 54% for the PVA group and 100%, 93%, and 58% for the ME-based TAE, which are equivalent to the experience reported by Maluccio et al. with TAE (84%, 66%, and 51% of patient survival at 12, 24, and 36 months) [18]. The robust results obtained by our group might be explained by the high proportion of BCLC A patients included in our cohort (36% of individuals in the PVA group and 25% of individuals in ME group). This concept was reinforced by a recent publication from our group, showing that BCLC A patients submitted to TAE had superior survival rates when compared with BCLC B patients exposed to the same treatment [19]. Takayasu et al. also have demonstrated longer survival rates in cirrhotic patients with preserved liver function [20]. In our study, an acceptable rate of tumor response was also found based on mRECIST criteria. Complete and partial response rates were identified, respectively, in 12.5% and 47.9% for patients treated with PVA and 21.9% and 56.3% for patients submitted to ME-based TAE, which are concordant with previous studies that have evaluated mRECIST as a prognostic marker [21, 22]. These findings support the idea that an adequate rate of tumor necrosis can be achieved with TAE, as long as a standardized protocol is followed.

Postembolization syndrome is related to tumor ischemia and to the local inflammatory response. There is a scarcity of data in the literature comparing the onset of side effects between different embolizing agents. Leung et al. were the first authors to establish a standardized PES score by applying the SOGC, in which almost 2/3 of the patients who underwent TACE developed PES (61.4%) [23]. In our cohort, there was no early death and the PES rate was lower than what has been reported before, suggesting that TAE induces fewer side effects [4, 6]. Interestingly, embolization with ME was associated with higher rate of PES (56.2% in the ME and 29.1% in PVA, *P* = 0.02), which shows that PVA is at least as safe as ME to perform TAE.

Another main aspect to highlight is that PVA-TAE is less costly than ME-TAE. The estimated cost per procedure in our hospital is of US$ 2000 for TAE with PVA and of US$ 2300 for TAE with ME. As in both groups the mean number of TAE procedures was similar (2.1 in the ME group and 2.3 in PVA group, *P* = 0.19), we believe that PVA represents an interesting alternative for locoregional treatment with fewer side effects and lower associated costs.

The retrospective nature of our study is one of the main limitations. Moreover, no other embolic material was tested, which limits comparisons. In addition to that, our study population sample was relatively small and the choice for the embolizing agent was not randomized. Thus, larger randomized studies are necessary in order to validate our findings.

## 5. Conclusions

In conclusion, TAE with ME was associated with more side effects when compared with PVA-based TAE, without any significant gain in survival, and also associated with an increased cost. Further studies are needed to compare not only the efficiency, but also the cost-effectiveness of each particular embolizing agent.

## Figures and Tables

**Table 1:** Postembolization syndrome according to the Southwest Oncology Group Toxicity.

Category	Grade				
	0	1	2	3	4
Pain	No	Use of nonnarcotics	Oral narcotics	Parenteral	Untreatable
Nausea	No	Oral intake ok	Reduction in oral intake	No intake	Parenteral
Vomits	No	1/24 h	2 to 5/24 h	6 to 10/24 h	More than 24 h
Temperature	No	37.1–38.0°C	38.1–40.0°C	Above 40.0°C	Fever and hypotension

**Table 2:** Baseline demographics and tumoral characteristics.

Characteristics	PVA (*n* = 48)	ME (*n* = 32)	*P* value
Age (years)	61 ± 8.3	60.4 ± 9.6	0.75
Gender (male)	58.3%	81.3%	0.06
Caucasians (%)	93%	99%	0.28
Body mass index (kg/m^2^)	26.1 ± 4.2	26.4 ± 4.4	0.76
HCV positive (%)	82.2%	74.2%	0.58
HCVRNA PCR qualit. (%)	90.0%	81.8%	0.60
Alcohol induced cirrhosis (%)	34.8%	34.5%	1.0
BCLC (%)			
A	36.0	25.0	0.36
B	60.0	75.0	
C	3.3	0	
Child-Pugh (%)			
A	43.8	59.4	0.25
B	50.0	31.3	
C	6.3	9.4	
MELD	12.4	14.3	0.12
Performance status (0)	93.8%	84.4%	0.26
Aspartate transaminase (U/L)	36.6	37.6	0.85
Alanine transaminase (U/L)	62.3	79.7	0.85
Gamma-glutamyl transferase (U/L)	134.9	117.2	0.48
Platelets (×1000/UL)	100.7	115.6	0.25
Prothrombin time (%)	72.9	76.9	0.26
Albumin (U/L)	3.25	3.46	0.22
Total bilirubin (mg/dL)	1.49	1.2	0.08
Alpha-fetoprotein (ng/mL)	(44) 2747	(28) 1027	0.22
Creatinine	0.93	0.95	0.72
CTMD	65.0%	51.0%	0.34
Tumor size (cm)	4.7	5.4	0.11
Number of tumors			
1	66.7%	25.0%	0.49
<3	29.2%	62.5%	0.48
≥3	4.2%	12.5%	0.48
Number of transarterial embolizations	2.3	2.1	0.19

PVA: polyvinyl alcohol; ME: microspheres; HCV, hepatitis C virus; HCVRNA PCR qualit.: C-reactive protein qualitative RNA hepatitis C virus; BCLC: Barcelona Clinic Liver Cancer; MELD: Model for End-Stage Liver Disease; CTMD: computed tomography multidetector.

**Table 3:** Response rates according to mRECIST (modified Response Evaluation Criteria in Solid Tumors).

	CR	PR	PD	SD
PVA (*n* = 48)	6 (12.5%)	23 (47.9%)	8 (16.7%)	11 (22.9%)
ME (*n* = 32)	7 (21.9%)	18 (56.3%)	3 (9.3%)	4 (12.5%)

CR: complete response; PR: partial response; PD: progressive disease; SD: stable disease; PVA: polyvinyl alcohol; ME: microspheres.

**Table 4:** Comparison of postembolization syndrome rates in patients treated with PVA or ME.

	0	1	2	3	4	5	>5
PVA (*n* = 48)	26 (54.2%)	8 (16.7%)	4 (8.3%)	6 (12.5%)	0	3 (6.3%)	1 (2.0%)
ME (*n* = 32)	11 (34.4%)	3 (9.4%)	11 (34.4%)	3 (9.4%)	3 (9.4%)	1 (3.0%)	0

PVA: polyvinyl alcohol; ME: microspheres.

## References

[1] Forner A., Llovet J. M., Bruix J. (2012). Hepatocellular carcinoma. *The Lancet*.

[2] El-Serag H. B. (2011). Hepatocellular carcinoma. *The New England Journal of Medicine*.

[3] Sangro B. (2014). Survival benefit with intraarterial techniques in hepatocellular carcinoma. *Gastroenterologia y Hepatologia*.

[4] Llovet J. M., Real M. I., Montaña X. (2002). Arterial embolisation or chemoembolisation versus symptomatic treatment in patients with unresectable hepatocellular carcinoma: a randomised controlled trial. *The Lancet*.

[5] Lammer J., Malagari K., Vogl T. (2010). Prospective randomized study of doxorubicin-eluting-bead embolization in the treatment of hepatocellular carcinoma: results of the PRECISION v study. *CardioVascular and Interventional Radiology*.

[6] Meyer T., Kirkwood A., Roughton M. (2013). A randomised phase II/III trial of 3-weekly cisplatin-based sequential transarterial chemoembolisation vs embolisation alone for hepatocellular carcinoma. *British Journal of Cancer*.

[7] Osuga K., Maeda N., Higashihara H. (2012). Current status of embolic agents for liver tumor embolization. *International Journal of Clinical Oncology*.

[8] Huppert P. (2011). Current concepts in transarterial chemoembolization of hepatocellular carcinoma. *Abdominal Imaging*.

[9] Tsochatzis E. A., Fatourou E., O'Beirne J., Meyer T., Burroughs A. K. (2014). Transarterial chemoembolization and bland embolization for hepatocellular carcinoma. *World Journal of Gastroenterology*.

[10] Tsochatzis E. A., Germani G., Burroughs A. K. (2010). Transarterial chemoembolization, transarterial chemotherapy, and intra-arterial chemotherapy for hepatocellular carcinoma treatment. *Seminars in Oncology*.

[11] Tsochatzis E. A., Fatourou E. M., Triantos C. K., Burroughs A. K. (2013). Transarterial therapies for hepatocellular carcinoma. *Recent Results in Cancer Research*.

[12] Malagari K., Pomoni M., Kelekis A. (2010). Prospective randomized comparison of chemoembolization with doxorubicin-eluting beads and bland embolization with BeadBlock for hepatocellular carcinoma. *CardioVascular and Interventional Radiology*.

[13] Xie Z.-B., Ma L., Wang X.-B. (2014). Transarterial embolization with or without chemotherapy for advanced hepatocellular carcinoma: a systematic review. *Tumour Biology*.

[14] European Association for the Study of the Liver (2012). EASL-EORTC clinical practice guidelines: management of hepatocellular carcinoma. *Journal of Hepatology*.

[15] Bruix J., Sherman M. (2011). Management of hepatocellular carcinoma: an update. *Hepatology*.

[16] Kocyigit A., Dicle O., Goktay Y., Astarcioglu I. (2014). The effect of using different embolic agents on survival in transarterial chemoembolization of hepatocellular carcinoma: gelfoam versus polyvinyl alcohol. *Diagnostic and Interventional Radiology*.

[17] Brown D. B., Pilgram T. K., Darcy M. D. (2005). Hepatic arterial chemoembolization for hepatocellular carcinoma: comparison of survival rates with different embolic agents. *Journal of Vascular and Interventional Radiology*.

[18] Maluccio M. A., Covey A. M., Porat L. B. (2008). Transcatheter arterial embolization with only particles for the treatment of unresectable hepatocellular carcinoma. *Journal of Vascular and Interventional Radiology*.

[19] Scaffaro L. A., Stella S. F., Alvares-Da-Silva M. R., Kruel C. D. (2015). Survival rates according to barcelona clinic liver cancer sub-staging system after transarterial embolization for intermediate hepatocellular carcinoma. *World Journal of Hepatology*.

[20] Takayasu K., Arii S., Ikai I. (2006). Prospective cohort study of transarterial chemoembolization for unresectable hepatocellular carcinoma in 8510 patients. *Gastroenterology*.

[21] Kim D. J., Clark P. J., Heimbach J. (2014). Recurrence of hepatocellular carcinoma: importance of mRECIST response to chemoembolization and tumor size. *American Journal of Transplantation*.

[22] Kuo Y.-C., Kohi M. P., Naeger D. M. (2013). Efficacy of TACE in TIPS patients: comparison of treatment response to chemoembolization for hepatocellular carcinoma in patients with and without a transjugular intrahepatic portosystemic shunt. *CardioVascular and Interventional Radiology*.

[23] Leung D. A., Goin J. E., Sickles C., Raskay B. J., Soulen M. C. (2001). Determinants of postembolization syndrome after hepatic chemoembolization. *Journal of Vascular and Interventional Radiology*.

